# A non-scalpel technique for minimally invasive surgery: percutaneously looped thread transection of the transverse carpal ligament

**DOI:** 10.1007/s11552-014-9656-4

**Published:** 2014-06-06

**Authors:** Danqing Guo, Yu Tang, Yizheng Ji, Tiansheng Sun, Joseph Guo, Danzhu Guo

**Affiliations:** 1 Department of Pain and Rehab Medicine, BayCare Clinic, 164 N. Broadway, Green Bay, WI 54303 USA; 2 Department of Orthopedic Surgery, Military General Hospital of Beijing, 5 Nan Men Cang St., East District, 100700 Beijing, China; 3 Ridge & Crest Company, 733 Ridgecrest St., Monterey Park, CA 91754 USA

**Keywords:** Transverse carpal ligament, Carpal tunnel release, Minimally invasive surgery, Carpal tunnel syndrome, Percutaneous procedure, Ultrasound-guided procedure, Percutaneous technique, Thread transection, Thread dividing

## Abstract

**Purpose:**

This study aims to develop an alternate technique for improving the surgical procedure of carpal tunnel release.

**Method:**

The transverse carpal ligament is transected by utilizing a piece of thread looped percutaneously under the visualization of ultrasound. The procedure, the thread carpal tunnel release (TCTR), was performed on 34 hands of 20 patients. Self-administrated Levine-Katz questionnaire was used for assessing the symptom severity and functional status of the outcomes.

**Results:**

TCTR was performed in each case with no unintended consequences. The average duration for a procedure was 7 min, excluding time of preparation. Significant improvements in subjective sensibility were reported within 24 h, and sleep quality improved for all cases. There were no postoperative complications. The scores of questionnaire 3 months postoperatively were comparable to the literature controls.

**Conclusion:**

TCTR is a safe and effective minimally invasive surgery performed under local anesthesia in a clinic-based procedure room and results in only one-needle entrance point at the wrist and one-needle exit point in the palm. The feature of the procedure includes the potentials of reduced risk of iatrogenic injury, reduced surgical cost, and reduced patient recovery time. The study has shown encouraging promise for optimizing the technique of carpal tunnel release, and more clinical trials are necessary to confirm the findings.

## Introduction

### Background

Carpal tunnel syndrome (CTS) is a common condition in the USA, with a prevalence of 3.7 % [24], and over 500,000 patients undergo carpal tunnel release (CTR) each year [7, 12]. CTS has ranked second in leading work time loss diagnoses [5, 10], and the estimated economic cost of CTR is up to $2.8 billion annually [15, 23].

As the most commonly performed surgical procedure in the treatment of CTS, open carpal tunnel release (OCTR) produces reliable symptom relief. OCTR requires an incision on the palm about 1 or 2 in. in length. Through this incision, the skin and subcutaneous tissue are divided, followed by the palmar fascia, and ultimately, the transverse carpal ligament (TCL). However, the subcutaneous tissue, superficial palmar fascia, and in some cases, the palmaris brevis have to be incised to expose the TCL. Consequently, scar tenderness, pillar pain, weakness, and a delay in return to work are known to occasionally occur [19, 25].

The limitations of OCTR resulted in the development of endoscopic carpal tunnel release (ECTR) in the late 1980s. ECTR involves one or two smaller incisions (less than 0.5 in. each) through which instrumentation is introduced including a synovial elevator, probes, knives, and an endoscope used to visualize the underside of the TCL. Although ECTR results in a rapid return of strength and function, concerns remain about the risks of median nerve injury and incomplete release [19, 20, 29]. Other drawbacks of ECTR include a narrow view of the surgical field provided by the endoscopic probe, a steep learning curve, the high device cost, and the significant setup time and effort required [4, 9]. The average return-to-work time is 54 days for OCTR and 28 days for ECTR [28].

In recent years, the development of ultrasound-guided procedures has provided a new approach for CTR. Ultrasound allows the exploration of carpal tunnel anatomy with a wide field of view at high resolution. Its flexibility, widespread availability, low cost, and short learning curve make it an effective tool in the diagnosis and treatment of CTS [3, 17].

Ultrasound-guided CTR was first reported in 1997 [13, 21, 27]. Since then, many researchers have focused on percutaneous procedures using different dividing elements to transect the TCL because ultrasound provides satisfactory surgical visualization. The selected dividing elements include hook knife [26], angle knife [22], saw blade [3, 17], and needle tip [16, 18, 22]. One weakness of the percutaneous approaches is that these mini-tools require repetitive cutting motions to divide the TCL, which increases the risk of technical errors including iatrogenic injuries or incomplete release, especially for patients with a narrow gap between the median nerve and the ulnar artery.

Therefore, it is advantageous to use a dividing tool with a mechanism different than the scalpel, blade, or needle tip, enabling surgeons to transect the TCL safely and effectively in the most minimally invasive way possible.

### Advantages of Thread as a Dividing Tool

Many people intuitively realize that the frictional effect of a sliding thread can cause a finger cut. This effect is more moderate than the abrasive effect found when using, for example, a Gigli saw. The frictional dividing of soft tissue concentrates shearing forces into the targeted tissue, resulting in significantly less collateral damage to adjacent anatomic structures than can be caused by the plowing and cutting of abrasive dividing [8].

Thread can be used to divide soft tissue in minimally invasive surgeries due to its unique properties: The flexibility of thread enables it to be routed accurately along a designated path to form a loop around the targeted tissue to precisely control the transection. The nature of thread transecting ensures that tissue is divided only inside the loop of thread around the targeted tissue without injuring adjacent non-targeted tissues. Thread can be easily placed using a spinal needle with only a few punctures as entry and exit points for the thread.

For safe and effective use of a dividing thread, the precision of the looping or routing process is essential. Precise routing is achieved by utilizing the real-time guidance of ultrasound.

### Thread Transection of Transverse Carpal Ligament

Using a flexible and smooth thread as a means to divide the TCL was proposed in 2012 by one of the authors, a specialist in tribology, the science and engineering of interacting surfaces in relative motion, and then, the percutaneous procedure of thread transection of transverse carpal ligament, called the thread carpal tunnel release (TCTR), has been developed in the same year. Firstly, the procedure was performed on a cadaver hand, and the immediate opening of the access showed the completeness of the transverse carpal ligament division and no injury to other structures. Then, the feasibility study, described in a later section, was conducted on 34 hands in 20 patients.

This article introduces the operational details of TCTR and discusses the feasibility and characteristics of this technique. We also present some features of TCTR on the basis of technical and theoretical analyses. The preliminary result of outcomes was compared with available literature controls of open and endoscopic surgeries through Levine-Katz questionnaire [14].

## Patients and Methods

A clinical study was conducted in a tertiary hospital in Beijing, China, on 34 hands of 20 patients to verify the feasibility of the TCTR surgical procedure. The cases are summarized in Table 1. All patients of TCTR were asked at 3 months of follow-up to fill in the Levine-Katz questionnaire for assessing symptom severity and functional status of the outcomes.

**Table 1:** The cases of thread transection for carpal tunnel release

Case	Sex	Age	Hand under surgery	Anesthesia
1	Female	53	Right and left	General
2	Male	83	Right and left	General
3	Male	44	Right and left	Local
4	Female	94	Right only	Local
5	Female	54	Right and left	General
6	Male	48	Right and left	Local
7	Female	73	Left only	Local
8	Female	61	Left only	Local
9	Male	32	Right and left	Local
10	Female	55	Right and left	Local
11	Female	40	Right and left	Local
12	Male	22	Right and left	Local
13	Male	36	Left first, right after 2 months	Local
14	Female	65	Left only	Local
15	Female	59	Right and left	Local
16	Female	47	Right and left	Local
17	Male	48	Right only	Local
18	Male	51	Right only	Local
19	Female	44	Right and left	Local
20	Female	45	Right and left	Local

### Tools

The tools consisted of a musculoskeletal ultrasound machine; an 18-gauge, 90-mm-long spinal needle; a piece of dividing thread; a powered hand tool; and a protective tube. The dividing thread is GuoPercutaneousWire™ (Ridge & Crest Company, Monterey Park, CA), a medical grade thread, with friction coefficient of 0.22, made from non-bonded PET with surface modified by a softening process. Each end of the thread was stiffened by covering it with a PTFE tube, 0.5 mm in diameter and 95 mm in length. GuoPercutaneousWire™ can be used manually in the same way as Gigli saw, and it takes longer time than with the help of a hand tool. The powered hand tool, TWP II (Ridge & Crest Company, Monterey Park, CA), has the function to simulate the manual back-and-forth motion that occurs when alternately pulling on the ends of the thread. The thread was passed through a protective tube made of PTFE with 2 mm ID and 20 mm length. The tube was held at the point of thread entry to preclude the opening in the skin from enlarging during the dividing process. TWP II has a simple structure, and its cost is about one third of an oscillating saw. One of the authors, a tribologist who developed the thread and tool for TCTR, had financial interest in the devices.

**Fig. 1:**
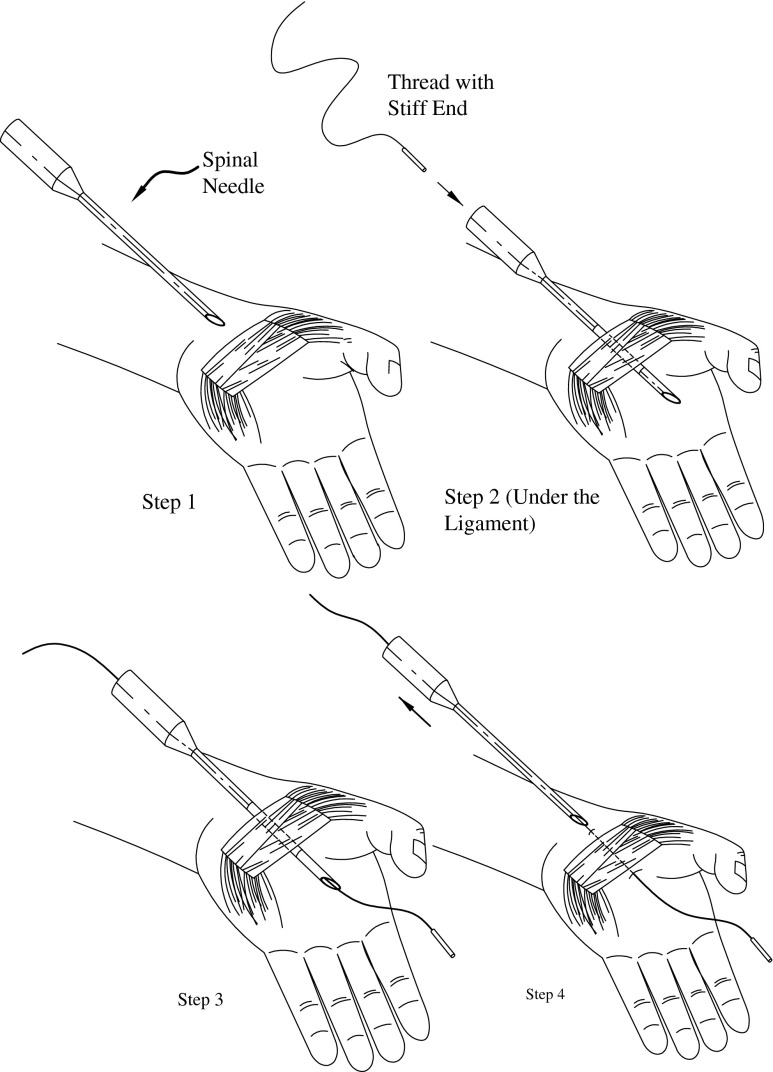
Procedure steps 1, 2, 3, and 4

**Fig. 2:**
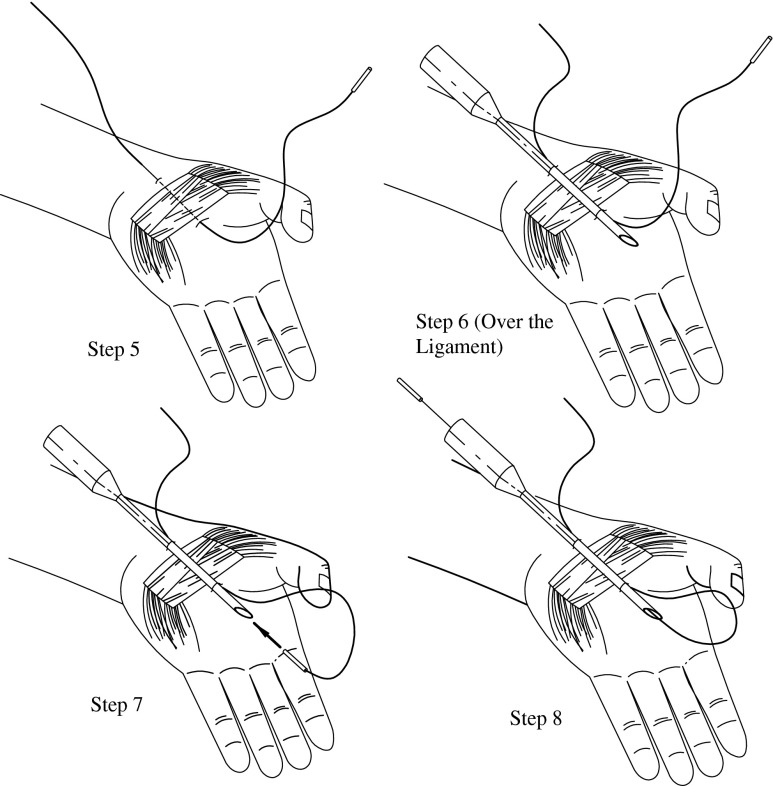
Procedure steps 5, 6, 7, and 8

**Fig. 3:**
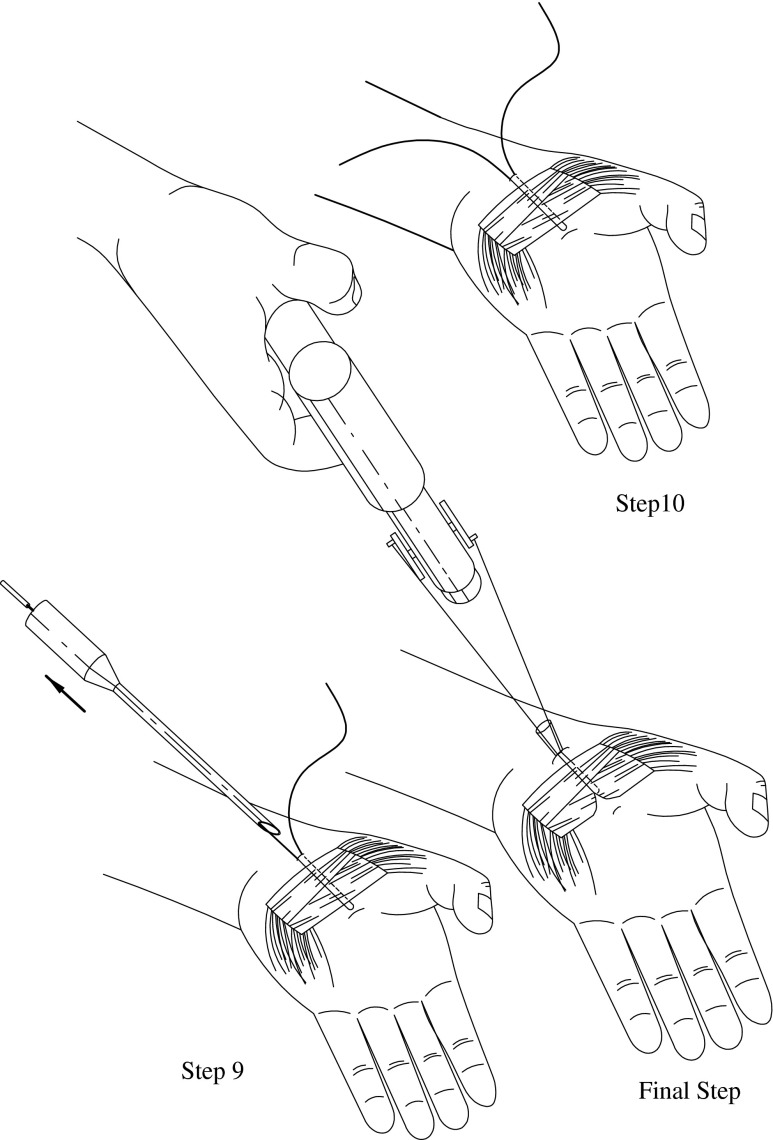
Procedure steps 9, 10, and 11

TCTR procedure included a preoperative ultrasound evaluation of volar wrist anatomy, local anesthesia, hydro-dissecting, and thread routing guided by ultrasound, confirmation of the looping, and transection of the TCL. Thread looping and transecting are shown graphically in Figs. 1, 2, and 3.

### Diagnosis and Procedure

All patients were Asian, and the average age was 52.7 years old (range 22 to 94). Twelve patients were female, and eight were male. Eight patients were employed prior to the procedure, and four patients were farmers. None of them had prior CTR surgery. Most patients had suffered from typical CTS for at least 12 months, and the conservative treatments for them failed. The symptoms included numbness and tingling in the median nerve sensory distribution, nocturnal worsening of numbness and tingling, and worsening of pain while holding or gripping. Their discomfort and pain scores varied between 8 and 10. Two of them also suffered from numbness and tingling in the little finger and in the hypothenar area. Most patients had thenar atrophy and abductor pollicis brevis weakness, and all of them were positive for Tinel’s sign and Phalen’s test. There were 15 patients with decreased two-point discrimination.

To confirm the diagnosis of CTS and to exclude other pathologic conditions, ultrasound evaluation of volar wrists was performed. It revealed that, in all cases, the cross-sectional area of the median nerve at the distal crease of the volar wrist was over 10 mm^2^, and the closest distance between the median nerve and the ulnar artery was 3 to 6 mm.

The procedures were performed under local anesthesia without conscious sedation for all the patients except the first two cases and case 5. The first two cases were under general anesthesia because of a conservative concern for patient safety and comfort, and case 5 requested general anesthesia because of the patient’s anxiety. When local anesthesia was employed, anesthetic was injected around the TCL during the process of hydro-dissecting, and patients were awake throughout the procedure.

#### Preoperative Evaluation

**Fig. 4:**
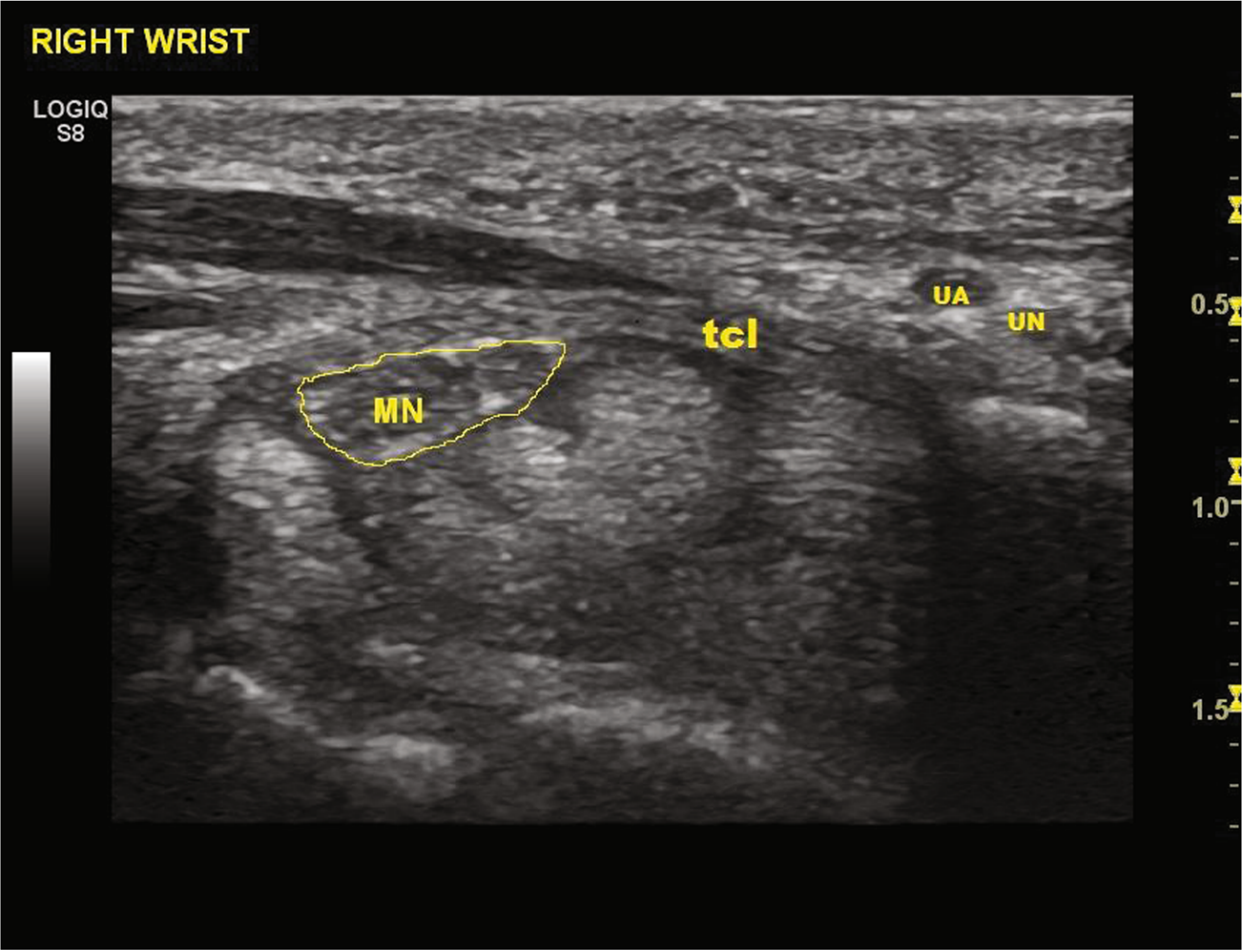
Ultrasound pre-examination of anatomy

The surgical field was prepared in standard fashion, and the patient’s hand was draped on a support pillow. A 12-MHz ultrasound transducer was utilized to evaluate the carpal tunnel and to locate the safe zone for dividing the TCL and to identify the median nerve, flexor tendons, proximal and distal margins of the TCL, bony marks of pisiform, tubercle of scaphoid, hook of hamate, trapezium, and superficial palmar arterial arch (SPA) (Fig. 4). Skin marks were made to identify the locations of the median nerve and the margins of the TCL, ulnar nerve, ulnar artery, and SPA. The needle entry point was marked 2 cm proximal to the distal crease of the volar wrist and between the median nerve and the ulnar artery. The exit point was marked at the intersection of Kaplan’s line and the radial aspect of the ring finger ray.

Each patient under local anesthesia was conscious during the procedure and cooperated with the surgeon to allow evaluation of the function of the hand, superficialis and profundus tendons, flexor pollicis longus, and thenar muscles. The three patients under general anesthesia were marked while awake prior to the administration of the anesthetic.

#### Hydro-Dissecting with Anesthetics Injection and Thread Looping

**Fig. 5:**
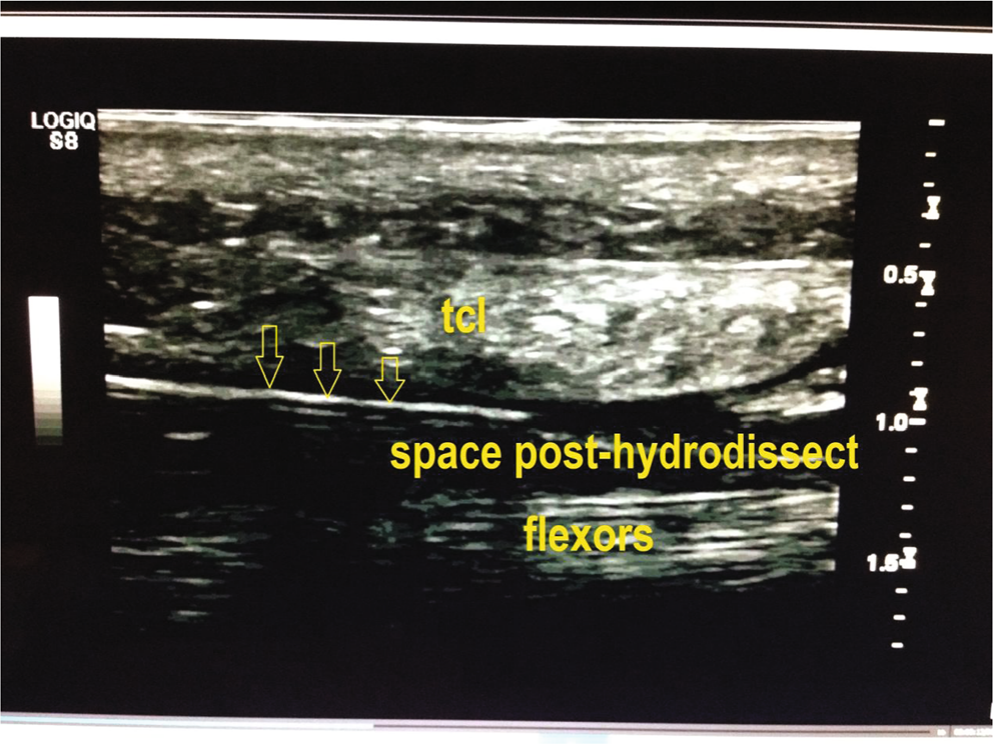
Hydro-dissecting at the low bounder of the ligament

After injecting 1 % lidocaine beneath the dermis at the entry and exit points, the needle was inserted into the subcutaneous layer and was advanced distally into the carpal tunnel within the safe zone between the median nerve and the ulnar artery. The needle was then advanced to the exit point. Simultaneously, the lidocaine solution was injected under real-time ultrasound observation to hydro-dissect the TCL from the median nerve (Fig. 5). Residual adhesion within the carpal tunnel, if any, was identified through active or passive motion of the fingers, allowing further release, if required, by additional hydro-dissecting. A total of 10 ml of 1 % lidocaine was used for each procedure.

**Fig. 6:**
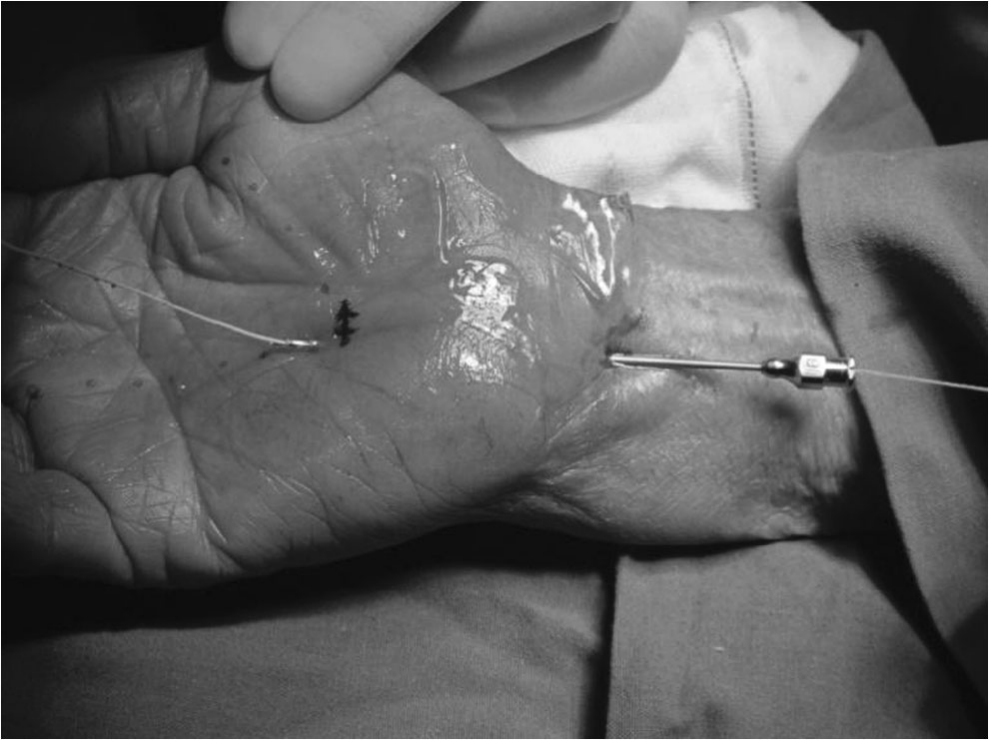
The thread passing through the needle

**Fig. 7:**
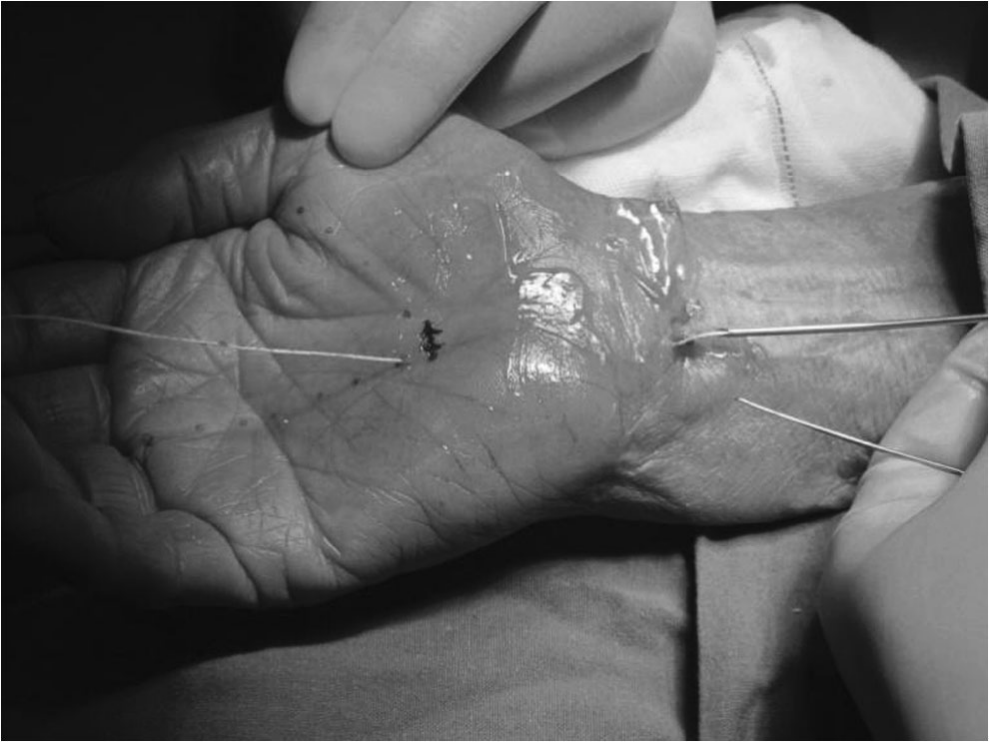
Pulling needle and leaving thread in place

To ensure that the surgical needle exited the hand at the desired location, either dorsal extension of the hand or a prebent needle or both were employed. Once the needle had exited the hand, a dividing thread was inserted into and through the needle (Fig. 6). The needle was then removed from the hand, leaving the dividing thread in place (Fig. 7).

**Fig. 8:**
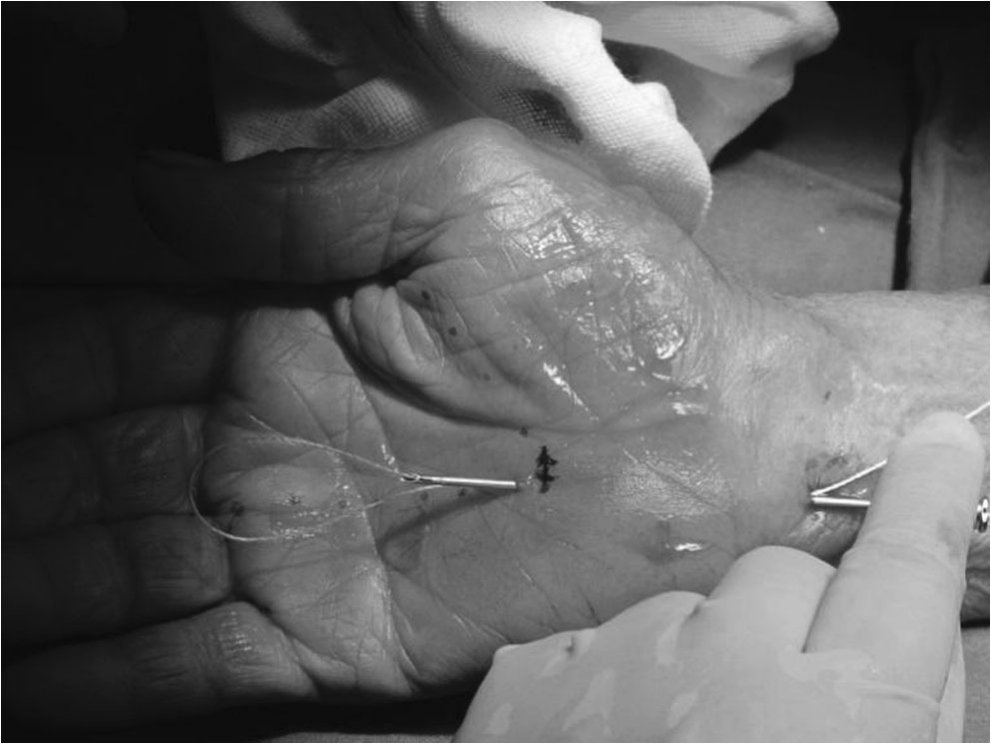
Inserting thread second time through the needle

The same needle was then inserted the second time into the same entry point at the proximal volar wrist and was advanced over the superficial surface of the TCL with hydro-dissecting to separate the interthenar fascia layer from the superficial surface of the TCL. The needle was guided to the same exit point at the palm. The thread emerging from the hand was then passed through the needle (Fig. 8). The needle was removed from the hand, leaving the dividing thread looped around the ligament.

The two ends of dividing thread were then placed through the protective tube and were linked to the motorized hand tool.

#### Confirming the Loop and Dividing the Ligament

**Fig. 9:**
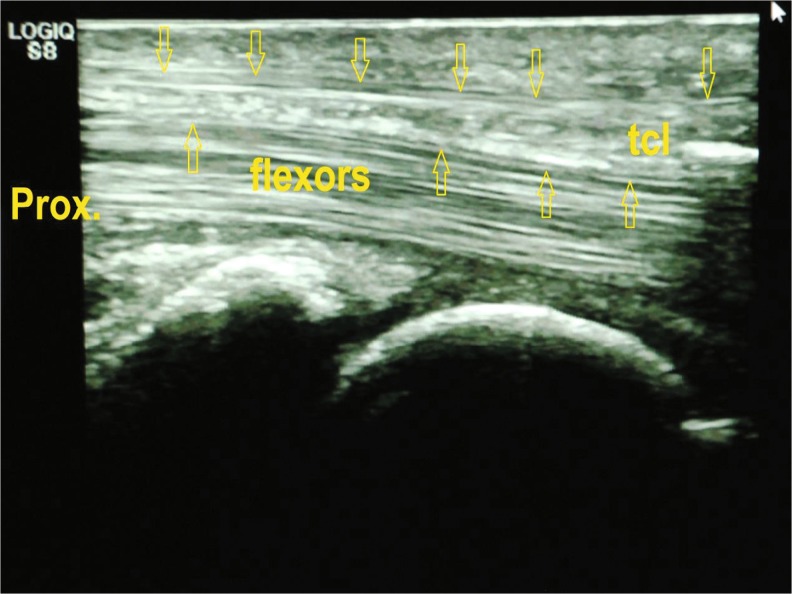
Thread in loop in ultrasound view

**Fig. 10:**
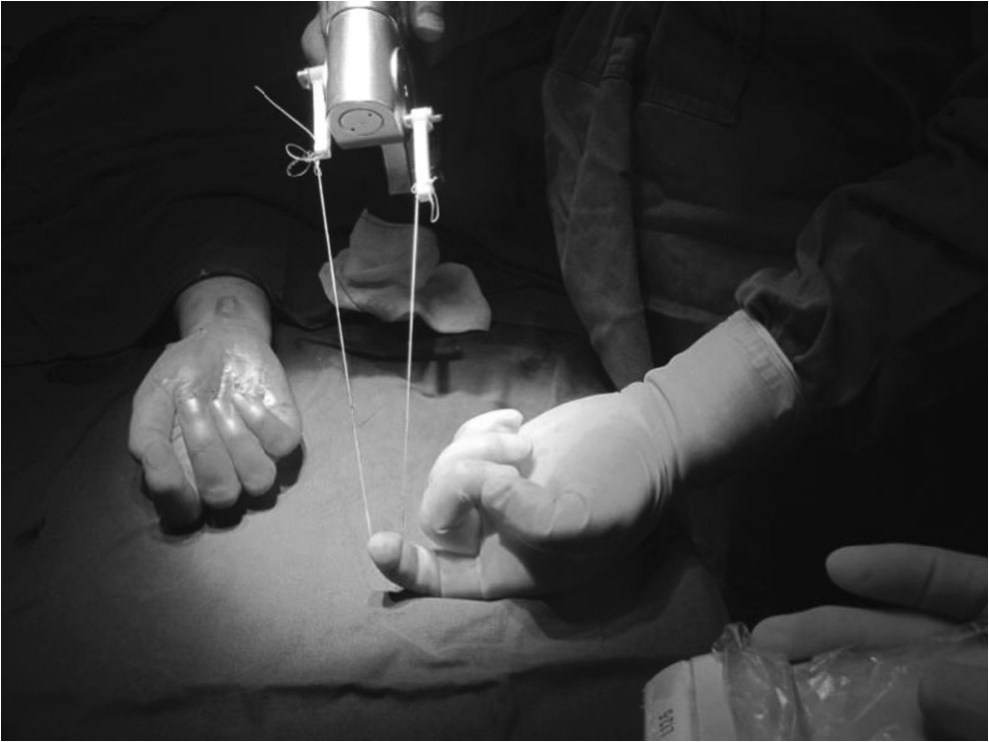
Thread was pulled out after dividing

The desired location of the inserted dividing thread along a looping path surrounding the TCL was verified by gently pulling on the thread and by using ultrasound to image the thread relative to the median nerve, SPA, and ulnar nerve (Fig. 9). After the correct looping was confirmed, the ligament transection was performed using the hand tool for 20 to 30 s. The thread was then removed from the hand through the initial entrance point at the wrist (Fig. 10). Finally, ultrasonic evaluation was employed to confirm that the TCL had been completely divided, and the median nerve, SPA, and flexor tendons remained intact.

We also manually performed dividing processes in recent cases, in the way same as operating a Gigli saw. Manual dividing with the help of two ring handles is controllable and effective, though it takes more than 1 min to complete transection of TCL. So, the hand tool is not necessary, but optional. We found that it was easier to manually perform dividing process through the exit point at the palm, instead of the point at the wrist, if manual dividing is selected.

## Results

All procedures were performed as described during the pilot clinical trial. For the most recent cases, the average duration of a procedure was 7 min, excluding the time of preparation, while the first one took 25 min to complete, and the second took 15 min.

A significant improvement in subjective sensibility was reported within 24 h, and sleep quality improved for all cases. For example, prior to the procedure, case 1 had severe numbness and tingling in both hands and woke up frequently at night. After the procedure, her pain score was reduced within hours to less than 4, and she slept well the same day. She returned to self-employed business in 10 days.

The case of the earliest return to work was a 40-year-old female patient who came back to her own business in 3 days, while a police officer patient, 22 years old, was back to work in 20 days. Four housewives with ages of 53 to 61 reported that they started household chores in 20 to 25 days, but a lady of 64 was in 60 days. Among eight patients employed before the procedure, the average return-to-work time for seven of them was 17.7 days (range 3 to 35 days). A construction worker did not return to work because he decided to change job, although he was satisfied with the outcome of procedure. The 6- and 12-month follow-ups for six early performed patients showed that there was no recurrence or functional difficulty.

Case 13, male patient at 36 years old, a mechanical technician, returned to work in 10 days after TCTR on his left hand. However, he had same symptoms of CTS on his other hand, right hand, 2 months later. After he tried conservative treatment with no help, he requested for the procedure of TCTR. Ultrasound evaluation confirmed the carpal tunnel syndrome with thickening of median nerve with cross-sectional area at distal wrist crease 14 mm^2^, and he subsequently had the procedure on his right hand. This time, he returned to work in 6 days.

Although there were no postoperative complications, case 2, who was 84 years old with a history of uncontrolled diabetes, ill-controlled hypertension, and degenerative cervical spondylosis, developed a swelling on his right wrist and hand with some limitation of flexion in his fingers 3 weeks after the procedure. Ultrasound revealed a normal superficial palmar arterial arch circulation and a normal sonographic median nerve image. Clinically, there was no infection, no signs of median nerve and ulnar nerve damage, and no deep venous thrombosis. Lab results of white blood cell count, erythrocyte sedimentation rate, and serum C-reactive protein did not suggest infection. EKG measurements revealed a third-degree A-V block with ventricular ectopic beats. The patient was treated by controlling hypertension and diabetes in consultation with an internal medicine specialist. After 3 days, the swelling in his hand had subsided, and normal function was recovered. Subsequent follow-up showed that the patient had good CTS relief with no complications.

The scores of self-administrated symptom severity and functional status (Levine-Katz questionnaire) from 16 validated questionnaires (18 were collected including two invalidated and two uncollected for less than 3 months) were presented in Table 2, comparing with the outcomes for open and endoscopic surgeries from literatures [1, 28].

**Table 2:** Scores of Levine-Katz questionnaire by 3 months and comparison

Study	Technique	Number of patients	Mean age	Symptom severity	Function status
Current	TCTR	16	52	1.4 ± 0.5	1.2 ± 0.3
Trumble et al. [28]	Open	72	56	1.9 ± 0.9	1.9 ± 0.9
Atroshi et al. [1]	Endoscopic	63	44	1.5 ± 0.5	1.3 ± 0.5

## Discussions

We are optimistic with the scores of symptom severity and functional status 3 months postoperatively, although the final result of the study is still in searching process, and the size of patients for the study is too small to have a strong statistical meaning. The scores of Levine-Katz questionnaire shows that the results of TCTR are slightly better than those of open and endoscopic surgeries, but for considering the limitation of the study, we would like to conclude that the outcomes of TCTR are at least similar to those of other techniques. Therefore, TCTR is safe and effective.

It is meaningful to further compare TCTR with OCTR and ECTR item-by-item, but at this moment, there has not yet been collected enough clinical data related to surgical outcomes and patient benefits. However, technical and theoretical analyses still play an important role in guiding the direction for a better technique of CTR.

Iatrogenic injuries that occasionally occur during surgery, such as damage to the median nerve, flexor tendons, or ulnar nerve, are often due to poor visualization of the surgical field and the long learning curve associated with OCTR and ECTR [19, 29]. However, ultrasound provides a high-quality image and real-time observation of the musculoskeletal structure and other soft tissues in the carpal tunnel area and immediate surroundings, including the TCL, flexor tendons, superficial palmar arterial arch, and median and ulnar nerves [4, 6, 11]. Additionally, the needle, the thread, and changes inside carpal tunnel due to hydro-dissecting are clearly visible using ultrasound. When routing is completed, the position of the loop of dividing thread can be verified relative to the TCL, the superficial palmar arterial arch, and other anatomical structures. If an incorrect thread path is indicated, the thread can be removed and immediately re-routed using the same procedure described. Lastly, the patient is awake during the entire procedure and can be asked to move fingers and thumb to evaluate hand function and check any possible damage in real time. The clear visualization and the ease of routing or re-routing the dividing thread could significantly reduce the risk of technique errors.

One of the advantages for reducing surgical risks is that if the surgeon encounters difficulties that require an early termination of TCTR procedure, it can be safely stopped at any step prior to the final dividing of the TCL. If further treatment or an alternate procedure is required, it can be safely scheduled for a later date.

Benson et al. found a striking difference between ECTR and OCTR in the rate of transient neuropraxia: 1.45 % for ECTR vs. 0.25 % for OCTR [2]. The higher rate for ECTR could be attributed to an iatrogenic injury to the median nerve caused by the insertion of a relatively large endoscopic cannula into the pressurized and diseased carpal tunnel, which was defined by Uchiyama et al., as the inherent weakness of ECTR [29, 30]. Uchiyama reviewed 311 cases of ECTR and found that the difficulties were encountered in 139 of 311 hands (44.7 %) during surgical process, and among those difficulties, 61 hands had the problem of tight access [30]. In contrast, an 18-gauge (or smaller) needle is used in TCTR for hydro-dissecting and routing, which avoids an immediate increase in carpal tunnel pressure and direct contact with the median nerve.

It is believed that minimizing the wrist and palm incisions benefits early return to activities of daily living or work. TCTR is minimally invasive, resulting in only two needle punctures. This maybe not only reduces the risk of infection but also results in less scar tenderness which benefits the postoperative recovery of patients. Additionally, selective dividing of the TCL with protection of the superficial palmar fasciae and interthenar fasciae decreases surgical trauma and is likely to minimize postoperative pain and weakness.

Although all pilot clinical trial procedures were performed by a hand surgeon with the assistance of an ultrasound specialist in an operating room, the percutaneous procedure was intentionally designed to be performed under local anesthesia in a clinic-based procedure room by a hand surgeon with an assistant, if the surgeon is capable of the use of ultrasound equipment and the interpretation of ultrasound results. Though ultrasound device is easily mastered, there exists a learning curve. An educational course on ultrasound is recommended if the practitioner does not routinely use ultrasound device in his clinical practice.

TCTR can lower the direct surgical cost to patients, mainly because it does not need a formal operating room and the help of an anesthesiologist, despite that the cost of an ultrasound device is additional. Based on the minimally invasive attributes discussed here, TCTR has the potential to lessen the social and economic burden of CTR through a shorter recovery time, potentially hastening a patient’s return to work.

TCTR may have limitations in cases where visualization using ultrasound is not sufficient or in complicated cases such as secondary carpal tunnel syndrome or with variant anatomies where OCTR may be indicated.

The study reported in this paper has shown that at least TCTR provides a safe and effective alternate to patients and that TCTR has the potential to further optimize the technique for CTR, but more clinical trials are necessary to confirm these findings.
